# Decreased Cerebral Creatine and N-Acetyl Aspartate Concentrations after Severe COVID-19 Infection: A Magnetic Resonance Spectroscopy Study

**DOI:** 10.3390/jcm13144128

**Published:** 2024-07-15

**Authors:** Jelena Ostojic, Dusko Kozic, Sergej Ostojic, Aleksandra DJ Ilic, Vladimir Galic, Jovan Matijasevic, Dusan Dragicevic, Otto Barak, Jasmina Boban

**Affiliations:** 1 Faculty of Medicine, University of Novi Sad, 21000 Novi Sad, Serbia; dusko.kozic@mf.uns.ac.rs (D.K.); aleksandra.dj.ilic@mf.uns.ac.rs (A.D.I.); vladimir.galic@mf.uns.ac.rs (V.G.); jovan.matijasevic@mf.uns.ac.rs (J.M.); otto.barak@mf.uns.ac.rs (O.B.); jasmina.boban@mf.uns.ac.rs (J.B.); 2 Faculty of Sport and Physical Education, University of Novi Sad, 21000 Novi Sad, Serbia; sergej.ostojic@chess.edu.rs; 3 Oncology Institute of Vojvodina, Diagnostic Imaging Center, 21204 Sremska Kamenica, Serbia; dragicevic.dusan@onk.ns.ac.rs

**Keywords:** magnetic resonance spectroscopy, COVID-19, creatine, N-acetylaspartate, absolute metabolite concentrations, neuroinflammation, noninvasive biomarkers

## Abstract

**Background/Objectives:** The aim of this study was to evaluate brain metabolism using MR spectroscopy (MRS) after recovery from Coronavirus disease (COVID-19) and to test the impact of disease severity on brain metabolites. **Methods:** We performed MRS on 81 individuals (45 males, 36 females, aged 40–60), who had normal MRI findings and had recovered from COVID-19, classifying them into mild (17), moderate (36), and severe (28) groups based on disease severity during the acute phase. The study employed two-dimensional spectroscopic imaging above the corpus callosum, focusing on choline (Cho), creatine (Cr), and N-acetylaspartate (NAA). We analyzed Cho/Cr and NAA/Cr ratios as well as absolute concentrations using water as an internal reference. **Results:** Results indicated that the Cho/Cr ratio was higher with increasing disease severity, while absolute Cho and NAA/Cr ratios showed no significant differences across the groups. Notably, absolute Cr and NAA levels were significantly lower in patients with severe disease. **Conclusions:** These findings suggest that the severity of COVID-19 during the acute phase is associated with significant changes in brain metabolism, marked by an increase in Cho/Cr ratios and a reduction in Cr and NAA levels, reflecting substantial metabolic alterations post-recovery.

## 1. Introduction

The coronavirus disease 2019 (COVID-19) has emerged as a complex viral syndrome with systemic implications, affecting multiple organ systems and ranging from mild symptoms to severe, life-threatening conditions [1]. Among its diverse complications, neurological manifestations have been frequently reported, attributed to direct viral effects, systemic inflammation, immune responses, hypoxia, and—occasionally—coincidental associations [2,3,4,5]. Notably, the virus (SARS-CoV-2) is known to invade the nervous system [6,7], with neuroimaging studies revealing both parenchymal signal abnormalities in severe cases [8] and widespread changes in brain microstructure among recovered individuals [9,10]. Recovery from COVID-19 has been linked to an increased risk of developing neurological or psychiatric conditions [11]. Studies on COVID-19 patients have highlighted critical brain injury biomarkers, such as neurofilament light chain (NfL), glial fibrillary acidic protein (GFAP), and matrix metalloproteinase-9 (MMP-9), which are essential in understanding the extent of neural damage. Elevated NfL levels in cerebrospinal fluid (CSF) and plasma during the acute phase have been associated with neurological symptoms indicative of acute axonal injury [2,12]. Similarly, increased GFAP levels reflect astrocyte damage and correlate with disease severity [13], while MMP-9 has been implicated in blood-brain barrier disruption, contributing to CNS damage and inflammation [14]. With the increasing interest in the involvement of neuroinflammation not only in COVID-19 but also in other viral infections and disorders not typically classified as primarily neuroinflammatory, such as neurodegenerative [15] and psychiatric disorders [16], there is a growing need for non-invasive methodologies for exploring these complex biological pathways. This broader perspective highlights the relevance of our study because by investigating the intricate pathways of neuroinflammation through technologies such as proton magnetic resonance spectroscopy (1H-MRS), we can gain insights that are applicable not only to infectious diseases but also to understanding the underlying mechanisms that may contribute to chronic neurological and psychiatric conditions. Proton MRS (1H-MRS) emerges as a non-invasive method for studying cerebral metabolism, providing paths for exploring biomarkers and potentially revealing neurometabolic shifts preceding observable structural and functional alterations. The measurability of metabolites, such as N-acetylaspartate (NAA), choline (Cho), creatine (Cr), and myo-inositol (MI), depends on various factors, including pulse sequence, spectral resolution, and signal-to-noise ratio (SNR). These metabolites serve as indicators of neuro-axonal viability, mitochondrial function, myelin degradation, and other critical aspects of brain metabolism [17,18]. In the context of neuroinflammation, these biomarkers can reveal significant insights into the underlying metabolic dynamics. While creatine (Cr) is often used as an internal reference due to its stability, its effectiveness may be limited in complex tissue pathologies, prompting the need for absolute quantification of metabolite levels to allow for robust intersubject comparisons. This study investigates whether COVID-19 affects non-invasive brain metabolite biomarkers and whether the severity of clinical presentation during the acute phase of COVID-19 infection influenced these metabolites. We selected the voxel locations—subcortical frontal white matter, anterior cingulate cortex (ACC), deep frontal white matter, and posterior cingulate cortex (PCC) based on their importance in neuroinflammation and technical considerations for optimal magnetic resonance spectroscopy [19,20,21]. We applied the method of quantifying brain metabolites by 1H-MRS using water as an internal standard. By using water as an internal reference, the quantification process becomes more standardized, reducing variability between measurements and across different studies. This technique is relatively simple and does not require additional calibration phantoms or external standards, enhancing its applicability in both clinical and research settings. A key advantage of using internal water in MRS studies is the elimination of factors and potential sources of error, such as RF homogeneity and coil loading because water and metabolite signals come from the same voxel and are acquired in essentially the same way [22]. Our findings reveal significant alterations in specific metabolites, reflecting the underlying neuroinflammation and offering new insights into the metabolic dynamics associated with COVID-19. This approach not only enhances our understanding of COVID-19’s neurological impact but also contributes to the broader field of neuroinflammatory research, emphasizing the importance of absolute metabolite quantification for reliable analysis.

## 2. Materials and Methods

### 2.1. Participant Recruitment and Demographic Information

We conducted magnetic resonance spectroscopy (MRS) at 3T on 81 individuals (40 males and 41 females, aged 40–60 years) 6–12 months after their recovery from COVID-19. These participants presented with various neurological symptoms associated with acute SARS-CoV-2 infection, including headaches; dizziness; disorders of smell or taste; and neurocognitive symptoms such as forgetfulness and issues with concentration and attention. None of the individuals participating in the study had any history of pre-existing neurological conditions prior to their SARS-CoV-2 infections. All individuals had normal MRI findings on conventional/structural MRI sequences. Demographic and clinical characteristics of the 81 study subjects are detailed in Table 1. Participants were divided into three groups based on the severity of their symptoms during the acute phase of the disease: mild (17), moderate (36), and severe (28). The mild group included outpatient-treated individuals with less severe clinical manifestations who exhibited at least one symptom of COVID-19 during the acute phase but did not require supplemental oxygen. The moderate group comprised hospitalized patients with a moderate-to-severe clinical presentation, who needed conventional oxygen support during the acute phase. The severe group consisted of hospitalized patients with severe clinical presentation, requiring higher levels of oxygen support during the acute phase, including high-flow nasal cannula (HFNC) therapy, non-invasive and invasive mechanical ventilation, or extracorporeal membrane oxygenation (ECMO). Participants were carefully selected based on the severity of their clinical presentations, with outpatient individuals sourced from the general population. Before enrollment, all eligible participants provided informed consent. Two neurologists independently examined the participants, and only those with normal neurological findings were included in the study. Exclusion criteria included clinically recognized and treated cognitive impairment; chronic degenerative, inflammatory, or vascular diseases of the central nervous system; confirmed malignant conditions; significant atherosclerotic disease of the carotid arteries diagnosed through neurosonological examination; and MRI findings indicating the presence of gray or white matter lesions, parenchymal inflammation, ischemia, hemorrhage, or brain tumors. On the other hand, patients with some other comorbidities (arterial hypertension, diabetes mellitus, chronic obstructive pulmonary disease, hyperlipidemia, hypothyroidism, etc.), who are present in our population in a high percentage, were not excluded from the study sample because they were undergoing optimal control and treatment of their health issues. We did not expect that to impact our results in this manuscript significantly.

### 2.2. Magnetic Resonance Imaging and Spectroscopy Procedures

**Figure 1:**
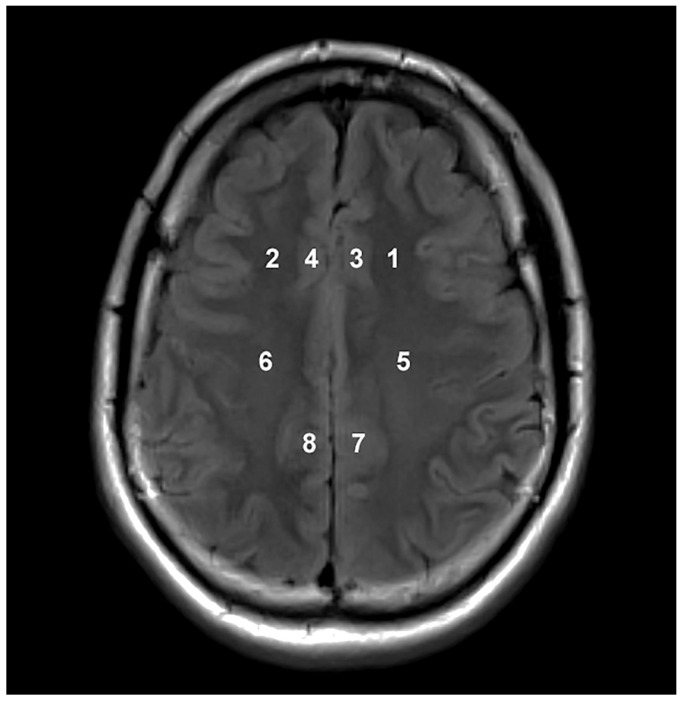
Axial FLAIR MR tomogram with typical position of 8 voxels: subcortical frontal white matter in the left hemisphere (1), subcortical frontal white matter in the right hemisphere (2), anterior cingulate cortex in the left hemisphere (3), anterior cingulate cortex in the right hemisphere (4), deep frontal white matter in the left hemisphere (5), deep frontal white matter in the right hemisphere (6), posterior cingulate cortex in the left hemisphere (7), posterior cingulate cortex in the right hemisphere (8).

**Figure 2:**
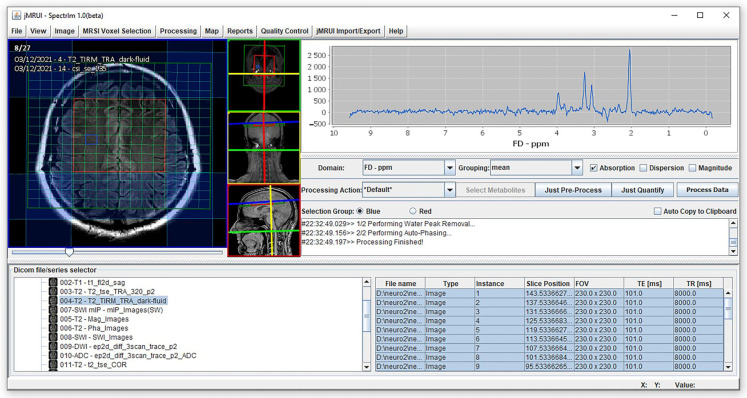
We selected each spectrum using SpectrIm because it allows for easy linkage of MRSI with morphological images. After selection, we transferred each spectrum to jMRUI and performed fitting using the AMARES technique. Figure 2 illustrates the voxel selection process for analysis. The voxel grid is displayed on a T2 morphological image, and the chosen voxel in the white matter of the right hemisphere corresponds to voxel number 6 in our study.

**Figure 3:**
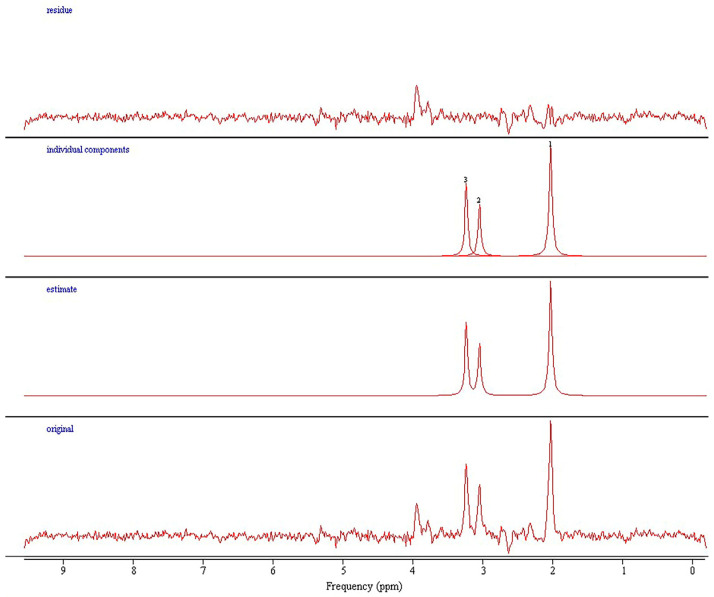
In the image, alongside the original spectrum, the results of fitting using the AMARES technique are also shown. The spectra are displayed in the frequency domain, with peaks labeled as 1, 2, and 3 corresponding to Cho, Cr, and NAA, respectively.

Magnetic resonance imaging and spectroscopy were performed using a 3T Siemens Trio scanner (Erlangen, Germany) equipped with a matrix head coil (receiver coil) in circularly polarized (CP) mode. Initially, we applied a routine MRI protocol for the brain to exclude any lesions and to precisely position the MRS voxel. This protocol included the following sequences: Sagittal T1-weighted spin-echo sequences with a repetition time (TR) of 440 ms, an echo time (TE) of 3.8 ms, and a slice thickness of 5 mm; axial T2-weighted turbo spin-echo (TSE) sequences with a TR/TE of 5150 ms/105 ms and a slice thickness of 5 mm; axial fluid-attenuated inversion recovery (FLAIR) sequences with a TR/TE of 8000 ms/101 ms and a slice thickness of 5 mm; diffusion-weighted imaging (DWI) with a TR/TE of 4100 ms/91 ms and a slice thickness of 5 mm; coronal T2-weighted TSE sequences with a TR/TE of 7150 ms/111 ms and a slice thickness of 5 mm. Water-suppressed proton two-dimensional spectroscopic imaging (CSI) datasets were acquired with point-resolved spectroscopy using a TR/TE of 1700/135 ms. The CSI slab had a field of view (FOV) of 160 mm × 160 mm × 160 mm, a voxel of interest (VOI) of 80 mm × 80 mm × 80 mm, and a thickness of 10 mm. It was positioned parallel to the axial images, immediately above the corpus callosum along the anterior–posterior commissure, to encompass the semioval white matter and the cortical gray matter. Voxel placement above the lateral ventricles ensures better homogeneity of the magnetic field, crucial for obtaining reliable and high-quality spectra. This positioning minimizes susceptibility artifacts and variations in magnetic field strength, enhancing the accuracy of the spectroscopic measurements. The number of phase-encoding steps (scan resolution) was 16 in all directions (R-L, A-P, and F-H), with 16 reconstructed spectra (interpolation resolution) in all directions, resulting in a VOI of 10 mm × 10 mm × 10 mm. Non-water-suppressed CSI data were obtained with the same geometric parameters (1 average) to provide an internal water reference for the absolute quantification of metabolites. A weighted phase-encoding scheme was applied, and interfering signal contributions from areas outside the VOI were suppressed by six saturation regions manually positioned along the margin of each VOI. The homogeneity of the magnetic field was optimized using an automatic, volume-selective shim provided by the manufacturer. Special care was taken to ensure that each region of interest was positioned identically for all participants to achieve the highest possible level of reproducibility. We implemented rigorous procedures to ensure consistency in voxel placement. Participants’ heads were positioned flat within the coil to avoid tilting or extension, ensuring uniform angulation for scans. Axial images aligned based on sagittal T1-weighted images were consistently placed just above the corpus callosum, providing a standardized basis for voxel placement. With a slice thickness of 3 mm, axial image acquisition enabled precise voxel selection containing identical anatomical structures across participants. Voxel placement was overseen by an experienced neuroradiologist specialized in MR spectroscopy, ensuring reproducibility and accuracy. We analyzed spectra from 8 individual voxels: subcortical frontal white matter in the left hemisphere (Location 1 in Figure 1), subcortical frontal white matter in the right hemisphere (2), anterior cingulate cortex in the left hemisphere (3), anterior cingulate cortex in the right hemisphere (4), deep frontal white matter in the left hemisphere (5), deep frontal white matter in the right hemisphere (6), posterior cingulate cortex in the left hemisphere (7), and posterior cingulate cortex in the right hemisphere (8). A total of 648 spectra were analyzed in this study. All acquired spectra met high quality standards in terms of signal-to-noise ratio and spectral resolution, and no data needed exclusion due to poorer quality or artifacts. Spectral fitting was performed using the Java-based MR user interface jMRUI software package version 7.0 with standard settings [23]. We utilized SpectrIm, a tool for the combined analysis of MR Spectroscopy and Imaging (Figure 2), which allows for the display of spectral grids on top of arbitrary MR image series. This tool facilitated the visualization, processing, and quantification of clinical MRS data, ensuring that voxel placement was verified and consistent. The experienced neuroradiologist also handled the selection of each voxel for analysis, ensuring consistency in anatomical structures included in each voxel across all participants. For the processing of metabolite spectra, the remaining water signal was removed using a Hankel Lanczos Singular Value Decomposition (HLSVD) filter, and amplitudes of Cho at 3.2 parts per million (ppm), Cr at 3.01 ppm, and NAA at 2.0 ppm were calculated with the Advanced Method for Accurate, Robust, and Efficient Spectral Fitting (AMARES) following established protocols (Figure 3) [24]. The amplitude of the water signal for each processed voxel was assessed from the scan without water suppression and used as an internal reference to calculate the absolute concentrations of the metabolites expressed in mM/kg. We calculated metabolite ratios of Cho/Cr, NAA/Cr, and Cho/NAA and absolute concentrations of Cho, Cr, and NAA. Mono-exponential spin-lattice and spin-spin relaxation were assumed, and published values of T1 and T2 relaxation times of water and respective metabolites measured at 3T in gray and white matter of healthy volunteers were used for relaxation corrections [25,26], the concentrations were calculated according to a previously described method [27].

### 2.3. Statistical Analyses

All statistical analyses were conducted using a uniform significance level of *p* < 0.05 to determine the statistical significance of all observed differences in metabolite ratios and absolute concentrations across brain locations. The sample size was determined by the total number of eligible patients available during the study period who met our inclusion criteria and passed the initial screening process. First, we tested for differences in gender and age across the entire sample and found no significant differences. We analyzed the normality of distribution for each group using skewness, kurtosis, and *p*-values to ensure the assumptions for applying statistical tests were met. These analyses confirmed the data distribution was appropriate and allowed us to proceed with further statistical analysis. Next, we employed a multivariate analysis of variance (MANOVA) to examine the overall differences among the three groups, which were categorized according to the severity of their disease symptoms. MANOVA tested the null hypothesis that there were no differences between groups classified by disease severity across all dependent variables (brain metabolites). The alternative hypothesis posited that there were differences between some severity groups on some metabolites. By considering multiple dependent variables simultaneously (metabolites), MANOVA accounted for the potential differences among them, providing a more comprehensive understanding of severity group differences. If MANOVA showed significant results, it indicated that differences existed between some severity groups on some metabolites, but it did not specify which groups and which metabolites were different. To identify specific differences, it was necessary to perform post-hoc *t*-tests between all possible pairs of groups for each metabolite individually. Upon obtaining significant results from MANOVA, we performed further detailed examinations using one-way analysis of variance (ANOVA) and Student’s *t*-tests to assess specific differences between the groups for all aforementioned metabolite ratios and absolute concentrations. We employed discriminant analysis (DA) as it allows for the simultaneous consideration of multiple variables, facilitating the differentiation between groups without necessitating multiple comparisons corrections [28]. DA tested the null hypothesis that there was no clearly defined boundary (multidimensional plane) between disease severity groups in the space of the examined metabolites, while the alternative hypothesis posited the existence of such a boundary. The analysis confirmed the existence of these boundaries, defined them, and characterized each subgroup relative to the boundary. It also determined the contribution of each metabolite to the characteristics of each disease severity group, allowing for the reduction of the variable space by excluding metabolites that did not significantly contribute to the disease severity group characteristics. Based on the multidimensional plane, the homogeneity of each disease severity group was determined relative to the identified characteristics. DA was utilized to identify which metabolite ratios and absolute concentrations significantly contributed to differentiating between disease severity groups across eight selected brain locations. Additionally, DA helped determine which brain locations significantly influenced the differences between groups in terms of the absolute concentrations of Cr and NAA. Using the coefficients from DA, we calculated the relative contribution (%) of each ratio and absolute concentration to the discrimination between disease severity groups across all eight locations.

## 3. Results

The sampling distributions for all observed metabolite ratios and absolute concentrations across all examined brain locations were approximately normal, as indicated by descriptive statistics and dispersion parameters. Using MANOVA, significant differences in metabolite ratios and absolute concentrations among groups classified by disease severity were detected across all brain locations. Further analysis with ANOVA revealed significant differences in Cho/Cr ratios at the frontal white matter (Locations 1 and 2) and anterior cingulate cortex (Locations 3 and 4); Cho/NAA ratios in the posterior cingulate cortex (Locations 7 and 8); absolute Cho concentrations in the anterior cingulate cortex and deep frontal white matter (Locations 4 and 6); and absolute concentrations of Cr and NAA across all observed brain regions. However, the NAA/Cr ratio did not show significant differences between disease severity groups in any of the studied locations (*p* > 0.05). Differences between all individual groups categorized by clinical severity were further investigated using a two-tailed *t*-test at all examined brain locations and are presented in Table 2, Table 3, Table 4, Table 5, Table 6, Table 7, Table 8 and Table 9. We displayed only absolute concentrations and metabolite ratios that significantly differ with a probability of *p* < 0.05. Through DA, we defined distinct metabolic profiles for each disease severity group at all locations and quantified the contributions of individual metabolites to these profiles. The three most influential metabolites or ratios for each brain location are presented in Table 10.

**Figure 4:**
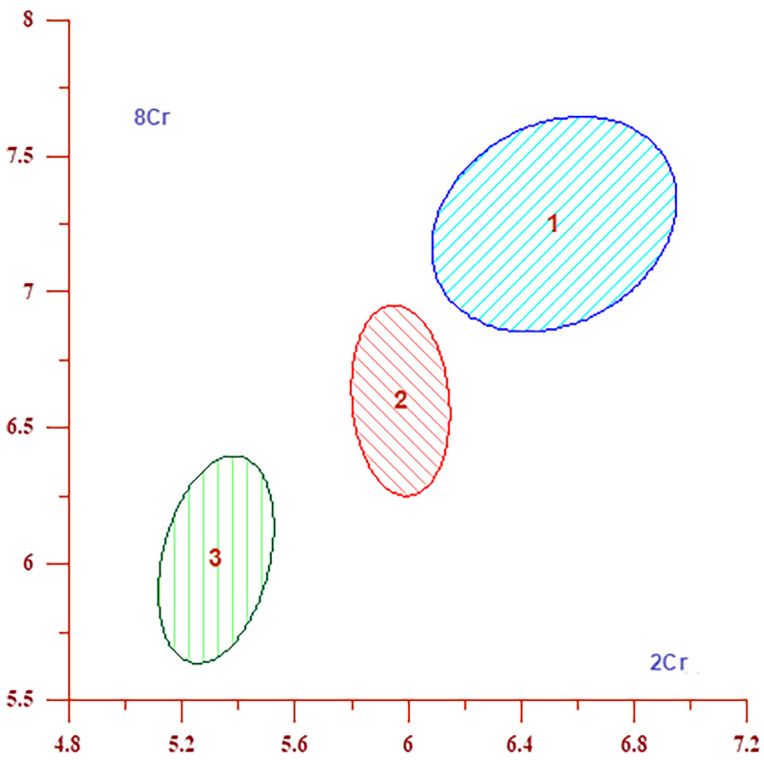
The graph displays confidence interval ellipses for Cr at brain Locations 2 and 8, where absolute Cr concentration contributes the most significantly to the differentiation between severity groups (Table 11). The graph indicates that the values of the absolute concentrations of Cr are higher in Group 1 (mild), lower in Group 2 (moderate), and lowest in Group 3 (severe) and that these groups are clearly separated.

**Figure 5:**
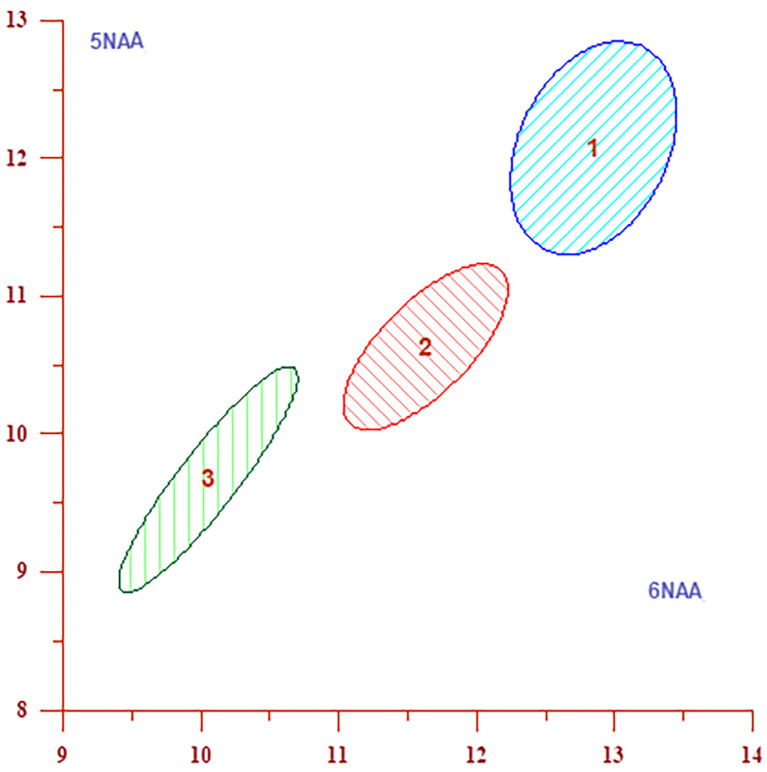
The graph presents confidence interval ellipses for the absolute concentrations of NAA at Locations 6 and 5, which play a key role in differentiating between severity groups, as indicated in Table 12. The graph reveals that NAA levels are highest in Group 1 (mild), reduced in Group 2 (moderate), and lowest in Group 3 (severe), clearly delineating these groups.

According to Table 10 and Table 11, the absolute concentration of Cr is lower in the severe group compared to both the moderate and mild groups at six out of eight studied locations. This observation is particularly pronounced in the subcortical frontal white matter of both the left (Location 1) and right hemispheres (Location 2), the deep frontal white matter of the right hemisphere (Location 6), and the posterior cingulate cortex of both hemispheres (Locations 7 and 8). At these specific locations, the absolute Cr concentration plays a substantial role in differentiating groups according to the severity of clinical presentations, more so than any other measured concentrations or metabolite ratios, as evidenced by Table 10. The DA results, presented in Table 11 and Table 12 and visually represented in Figure 4 and Figure 5, clearly demonstrate significant interactions between disease severity and specific brain locations for both Cr and NAA metabolite concentrations. These findings highlight the critical regions in the brain where metabolite changes are most pronounced in relation to the severity of COVID-19 symptoms during the acute phase. Table 11 presents the absolute concentrations of Cr for all locations, ranked by their contribution to distinguishing between severity groups. We have identified that Locations 2 (subcortical frontal white matter in the right hemisphere), 8 (cingulate cortex in the right hemisphere), and 7 (cingulate cortex in the left hemisphere) are key in differentiating between severity groups for Cr concentrations. Calculation of homogeneity (Table 11) revealed that for the absolute concentration of Cr, 15 out of 17 participants exhibited characteristics of the mild group, or 88.24%; 23 out of 36 participants exhibited characteristics of the moderate group, accounting for 63.89%; and 20 out of 28 participants (71.43%) exhibited characteristics of the severe group. The results from Table 11 are also visually represented in Figure 4, which displays confidence ellipses. These ellipses are generated by plotting confidence intervals for absolute Cr concentrations at Locations 2 and 8 the key locations that contribute to differentiating between severity groups for Cr. Levels of NAA were significantly lower in severe cases compared to those classified as mild and moderate. As shown in Table 10, the absolute concentration of NAA at seven of the eight locations is among the three variables most significant in differentiating between severity groups. Table 12 displays the absolute NAA concentrations across all locations by contribution level, revealing that NAA more significantly discriminates between mild, moderate, and severe groups, primarily in white matter Locations 6 (deep frontal white matter in the right hemisphere), 5 (deep frontal white matter in the left hemisphere), and 2 (subcortical frontal white matter in the right hemisphere). Complementing this, Figure 5 illustrates the data through confidence ellipses that are based on confidence intervals for NAA at locations 6 and 5. These locations are pivotal in distinguishing between the severity groups for NAA. The homogeneity data (Table 12) for the absolute concentration of NAA indicates that 13 out of 17 (76.47%) individuals in the mild group exhibit characteristics of this group, 19 out of 36 (52.78%) individuals in the moderate group exhibit characteristics of this group, and 20 out of 28 (71.43%) individuals in the severe group exhibit characteristics of the severe group. While the ANOVA did not reveal statistical significance in the differences in absolute Cho concentrations, subsequent *t*-tests identified significantly lower Cho levels in the severe group compared to the mild group at specific locations (Table 2, Table 3, Table 4, Table 5, Table 6, Table 7, Table 8 and Table 9), and discriminant analysis confirmed its significant role in differentiating disease severity at Location 3 (anterior cingulate cortex, right hemisphere), as demonstrated in Table 10. The Cho/Cr ratio was significantly higher in the severe group compared to mild and moderate groups at several locations (Table 2, Table 3, Table 4, Table 5, Table 6, Table 7, Table 8 and Table 9). Cho/Cr significantly contributes to distinguishing between groups only at Location 4, the anterior cingulate cortex in the right hemisphere. Cho/NAA ratios were significantly higher in severe versus mild cases at specific locations, making a significant contribution to distinguishing between severity groups at Locations 1, 3, 4 and 5.

## 4. Discussion

To the best of our knowledge, this is the first MRS study to examine normal-appearing brain parenchyma on MRI following COVID-19 infection of varying severity. While previous COVID-19 MRS studies predominantly focused on acute and hospitalized cases, encompassing a wide range of gross cerebral abnormalities, such as white matter hyperintensities and cerebrovascular events [6,7,8,9,10], none have quantitatively compared MRS data across subjects with varying disease severities. MRS, an advanced imaging technique used in conjunction with MRI, provides complementary, non-invasive insights into the biochemical composition of imaged tissues. Prominent signals in the 1H spectrum include NAA, Cr, and Cho. Although frequently used as an internal reference, Cr concentration can vary with neurological conditions and diseases, necessitating the calculation of absolute Cr concentrations [29]. Within the CNS, Cr plays a crucial role in energy metabolism and is essential for maintaining membrane potentials, ion gradients, and calcium homeostasis [30]. It also acts as a potent antioxidant, effectively neutralizing reactive oxygen species (ROS) [31], and exhibits neuroprotective effects in rodent models of ischemia/anoxia [32]. Our previous research indicated that six months of Cr supplementation could improve tissue bioenergetics and alleviate symptoms of post-COVID-19 fatigue syndrome [33]. Furthermore, Cr might protect the CNS in neurodegenerative diseases such as Parkinson’s or Huntington’s by providing cellular energy support [34]. Our findings indicate that patients with severe COVID-19 who were hospitalized and required high levels of oxygen support during the acute phase had lower brain concentrations of Cr and NAA compared to those with mild and moderate symptoms. Similarly, individuals with moderate-to-severe symptoms requiring conventional oxygen support had lower brain concentrations of Cr and NAA than those not needing supplemental oxygen. A study on newborns within four days of birth linked decreased brain Cr concentration to outcomes ranging from normal/mild to severe/fatal, attributing this decrease to reduced cell density within neonatal encephalopathy groups compared to controls [35]. The need for respiratory support in the delivery room, a common practice in preterm infants, has been identified as a potential cause of brain injury, termed ventilation-induced brain injury (VIBI) [36,37], where even brief periods of high tidal volume ventilation can elevate early indicators of brain injury through systemic inflammatory responses and hemodynamic instability [37,38]. Therefore, a group of researchers investigated whether white matter MRS and diffusion tensor imaging (DTI) can detect brain injury resulting from mechanical ventilation in preterm lambs. The authors further clarified that the notable reductions in the absolute concentrations of Cr and Cho, as measured by MRS in lambs exposed to ventilation when compared to the control group, suggest a loss of tissue and a decrease in cell density [39]. On the other hand, a volumetric study involving 155 patients found significant variations in brain volumes that correspond with the severity of COVID-19. Predominantly, the atrophy affected the total and supratentorial grey matter, including the frontal and parietal lobes, as well as the right thalamus. The observed group differences remained significant even after excluding ICU-treated patients, leading the authors to discount the potential impact of mechanical ventilation or intensified drug therapy on the overall outcome. The authors propose that the neocortical damage arose as a consequence of COVID-19, potentially linked to the initial severity of the disease [40]. Another study analyzed post-COVID-19 patients with mild respiratory symptoms not requiring oxygen supplementation, hospitalization, or assisted ventilation reported a significant global reduction in cortical grey matter (CGM) volume, supporting the notion that brain changes are not solely attributable to mechanical ventilation [41]. The results of our study also mitigate the possibility that biochemical changes in the brain result from mechanical ventilation, as these changes are present even in the group of individuals with a moderate clinical presentation who only received conventional oxygen support during the acute phase. Cr is decreased in hypermetabolic states and across different neurodegenerative conditions [42,43]. One such condition is early Alzheimer’s disease, in which a decreased absolute concentration of Cr has also been identified [44]. This finding in our patients could possibly indicate an increased risk of dementia following severe COVID-19 infection. In our study, NAA peak is decreased in both gray and white matter following severe forms of COVID-19. NAA is synthesized in neurons and is considered a marker of neuronal density and function. The reduction of NAA levels in white matter indicates diffuse axonal loss or damage, while the decrease in gray matter suggests neuronal loss. Song et al. demonstrated the potential for SARS-CoV-2 to directly invade human brain tissue and cause neuronal death, providing a possible explanation for reduced NAA due to direct neuronal loss [45]. The results of our study offer stronger support for neuron loss after severe COVID-19 than volume measurements by MRI alone. The observation of low brain NAA following COVID-19 could potentially be linked to other underlying mechanisms: NAA is involved in mitochondrial metabolism in neurons. COVID-19 has been shown to affect mitochondrial function in various cell types, and such dysfunction in neurons could lead to decreased NAA production. This might reflect a broader impact of the virus on cellular energy metabolism. It has been established that COVID-19 can lead to mitochondrial dysfunction, potentially affecting various organs, including the brain [46]. Disturbances in mitochondrial function could influence NAA levels. Systemic inflammation, a hallmark of severe COVID-19, can also affect the brain. Inflammatory cytokines can cross the blood–brain barrier, potentially leading to a neuroinflammatory response that could disrupt neuronal function and metabolism, thereby affecting NAA levels. A bidirectional relationship has been identified between COVID-19 and psychiatric disorders, implicating inflammatory pathways that could affect neuronal integrity and NAA concentrations [47]. Oxidative stress has been implicated in COVID-19 pathophysiology and can lead to neuronal damage. The brain is particularly vulnerable to oxidative stress due to its high oxygen consumption and lipid-rich environment. This stress can damage neurons and lead to alterations in NAA. It was hypothesized that the pathogenesis of SARS-CoV-2 infection, including its neurological impact, involves oxidative stress mechanisms, potentially resulting in neuronal damage and alterations in NAA [48]. Studies have shown how the spike protein of SARS-CoV-2 can disrupt the blood–brain barrier in vitro, suggesting a mechanism by which the virus could indirectly lead to brain inflammation and neuronal damage, potentially affecting NAA levels [49]. Cho, as a metabolite, is a key component of phospholipids, serving as a precursor for the neurotransmitter acetylcholine and playing a crucial role in cell membrane integrity and signaling processes within the brain [50]. According to the findings of our study, there was no increase observed in the absolute concentration of Cho in the groups of participants with more severe clinical presentations compared to those with milder conditions. In fact, we even noted a decrease in choline levels at specific locations in the groups with more severe clinical presentations. Additionally, a low absolute concentration of Cho was measured in the magnetic resonance spectroscopy (MRS) examination of normal-appearing white matter (NAWM) in patients with MR imaging-negative multiple sclerosis (MS) [51]. The authors suggested that this might be attributed to an increased uptake of Cho from the free phase for cell membrane synthesis and speculated that it could signify the initiation of a healing process in these MS patients. In our study, the absolute concentrations of metabolites contribute more to the differences between disease severity groups compared to metabolite ratios: Cho/Cr, Cho/NAA, and NAA/Cr. It was found that the NAA/Cr ratio does not significantly differentiate between disease severity groups, attributed to the reduction in both metabolites. The Cho/Cr ratio is elevated in groups with more severe clinical presentations not due to an increase in Cho but rather to a decrease in Cr. Cho/NAA values rise with the severity of clinical manifestations, owing to diminished NAA, while the choline peak remains relatively stable, even indicating a trend towards a slight decrease. In previous studies comparing metabolite ratios to absolute quantification, it was found that metabolite ratios reduce the sample size requirements and increase statistical significance compared to cases of absolute quantification, particularly when the numerator and denominator shift in opposition in pathological conditions [29,52]. However, when the numerator and denominator move in the same direction—i.e., when both decrease, as in our study, or when both increase—calculating absolute concentrations proves to be favorable. Studies indicate that neurological effects of COVID-19 can persist long after the acute phase, making it important to study brain metabolism months after recovery. Research shows that cognitive deficits and fatigue can last for several months, with changes in brain metabolism detectable beyond the acute phase [53]. Our findings reveal statistically significant differences in brain metabolism between groups based on the severity of their acute phase symptoms, suggesting that the timing of MRS measurements did not obscure these differences. The 6–12-month post-recovery period is crucial for capturing the long-term effects of COVID-19, allowing us to observe chronic changes in brain metabolism that are not immediately apparent but are essential for understanding the virus’s prolonged impact on the central nervous system [54].

Our study has several limitations: The first limitation is that we lack MRS data from our participants before COVID-19, and we cannot rule out the possibility that they had altered metabolism prior to infection. The second limitation is that we did not investigate the myo-inositol peak (mI), which has been found to be elevated in some studies of neuroinflammation [18,55,56] while unchanged in others [57,58]. The third limitation is that we did not perform voxel segmentation, i.e., we did not eliminate the contribution of cerebrospinal fluid in the examined voxel. This may partly affect gray matter voxels but not white matter voxels, as the white matter voxels contained only white matter without cerebrospinal fluid and gray matter content. The entire MRI and MRS examination process, including voxel selection for analysis, could be significantly improved by applying cutting-edge techniques such as the 3D Dual-CycleGAN model to generate T1c, T2W, and FLAIR-like images from T1W images [59].

## 5. Conclusions

To conclude, the results of our study confirm that MRS can be a reliable technique for detecting the effects of COVID-19 infection on brain metabolites in long-term follow-up, with potential to reflect the severity of infection. Both in grey and white matter, the decrease in NAA suggests the neuronal loss and/or dysfunction following direct neuronal injury caused by virus per se or indirect neuronal loss caused by the neuroinflammatory processes triggered by systemic inflammation. One of the most interesting findings is the instability of Cr, with detected decrease reflecting the severity of the clinical condition possibly indicating an increased risk of neurological complications such as dementia following severe COVID-19 infection. Finally, we observed relatively stable or even decreasing Cho in the more severe clinical presentations potentially speaking in favor of neuroplasticity in observed voxels. The alterations in brain metabolites observed in our study may be attributed to a combination of direct viral effects, systemic inflammation, oxidative stress, mitochondrial dysfunction, and hypoxia. These mechanisms collectively may disrupt neuronal health and energy metabolism, leading to decreased levels of NAA and Cr, and altered Cho dynamics. Overall, these findings highlight the potential for significant long-term impacts on brain metabolites following COVID-19 infection, emphasizing the need for ongoing monitoring and further research into these effects.

## Figures and Tables

**Table 1:** Table with demographic and clinical data for study participants.

Description	Mild (*n* = 17)	Moderate (*n* = 36)	Severe (*n* = 28)
**Median Age (range)**	49 (40–58)	51 (42–60)	52.5 (45–60)
**Gender**			
Male	7	19	14
Female	10	17	14
**Weight average (range)—kg**	76.27 (53–105)	92 (60–125)	88 (51–120)
**BMI kg/m^2^ average (range)**	25.36 (19.2–33.9)	29.8 (20.7–40.8)	29.6 (18.7–45.7)
**Acute phase symptoms N (average duration—days, duration range—days)**			
Fever Malaise	16 (4.8; 1–4) 15 (12.1; 1–30)	26 (8.6; 3–16) 24 (23.5; 3–180)	25 (15.48; 3–60) 21 (25.2; 2–90)
Myalgia	15 (9.5; 1–40)	13 (34.5; 3–180)	15 (19.8; 5–60)
Arthralgia	13 (10.6; 2–30)	9 (36.6; 3–180)	11 (23.6; 5–60)
Nausea	8 (10.5; 1–30)	8 (5.7; 2–14)	11 (8.1; 2–15)
Vomiting	3 (5.3; 1–10)	5 (3; 1–7)	3 (7; 2–14)
Diarrhea	6 (5.7; 1–14)	10 (4.6; 2–9)	12 (8.1; 2–20)
Sore throat	6 (5.3; 1–10)	8 (5.6; 3–10)	10 (9.9; 3–15)
Cough	6 (13.6; 2–21)	16 (10.8; 3–30)	14 (14.7; 7–60)
Headache	15 (9.1; 2–21)	9 (8.1; 2–15)	13 (14.8; 7–60)
Dizziness	7 (4.8; 1–10)	9 (8.5; 3–30)	6 (9.3; 4–15)
Altered taste	12 (33.6; 5–120)	11 (15.6; 4–30)	8 (35.5; 7–210)
Altered sense of smell	10 (25.1; 5–90)	13 (14; 3–30)	8 (38.2; 7–210)
Loss of Appetite	5 (6.6; 2–14)	3 (10; 10–10)	10 (9.9; 3–15)
Limb tingling	2 (25; 10–40)	/	9 (77.1; 10–240)
**CRP in Acute phase (average range)**	/	86.3 (8.2–239.2)	145.6 (3.4–350)
**Comorbidities**			
Arterial hypertension	3	12	3
Diabetes	1	9	2
Lung disease (Asthma, CODP)	/	/	1
Hyperlipidemia	1	5	1
Hypothyroidism	3	3	/
Psychiatric disorder (depression)	/	1	/
Deep venous thrombosis	/	1	2

**Table 2:** The results of the paired-samples *t*-test comparing disease severity groups in the subcortical frontal white matter in the left hemisphere (Location 1).

AC (mM/kg)/Ratio	Groups		Mean		SD		*t*	*p*
Cho/Cr	mild	moderate	1.154	1.271	0.13	0.16	2.673	0.010
Cho/Cr	mild	severe	1.154	1.377	0.13	0.20	4.573	0.001
Cho/Cr	moderate	severe	1.271	1.377	0.16	0.20	2.417	0.019
Cho/NAA	mild	severe	0.666	0.739	0.11	0.12	2.077	0.044
Cr	mild	moderate	6.469	5.827	0.86	0.59	3.187	0.022
Cr	mild	severe	6.469	5.233	0.86	0.74	5.133	0.001
Cr	moderate	severe	5.827	5.233	0.59	0.74	3.590	0.009
NAA	mild	moderate	9.535	8.902	1.08	1.02	2.071	0.043
NAA	mild	severe	9.535	8.291	1.08	1.35	3.218	0.002
NAA	moderate	severe	8.902	8.291	1.02	1.35	2.063	0.043

AC absolute concentration, SD standard deviation.

**Table 3:** The results of the paired-samples *t*-test comparing disease severity groups in the subcortical frontal white matter in the right hemisphere (Location 2).

AC (mM/kg)/Ratio	Groups		Mean		SD		*t*	*p*
Cho/Cr	mild	moderate	1.181	1.284	0.16	0.17	2.078	0.043
Cho/Cr	mild	severe	1.181	1.390	0.16	0.16	4.304	0.012
Cho/Cr	moderate	severe	1.284	1.390	0.17	0.16	2.533	0.014
Cho/NAA	mild	severe	0.648	0.736	0.11	0.10	2.720	0.009
Cr	mild	moderate	6.517	5.973	0.87	0.52	2.838	0.046
Cr	mild	severe	6.517	5.321	0.87	0.54	5.733	0.001
Cr	moderate	severe	5.973	5.321	0.52	0.54	4.903	0.039
NAA	mild	severe	10.075	8.502	1.34	1.29	3.903	0.025
NAA	moderate	severe	9.384	8.502	1.19	1.29	2.829	0.026

AC absolute concentration, SD standard deviation.

**Table 4:** The results of the paired-samples *t*-test comparing disease severity groups in the anterior cingulate cortex in the left hemisphere (Location 3).

AC (mM/kg)/Ratio	Groups		Mean		SD		*t*	*p*
Cho/Cr	mild	severe	1.155	1.322	0.13	0.14	4.052	0.001
Cho/Cr	moderate	severe	1.225	1.322	0.17	0.14	2.412	0.019
Cho/NAA	mild	severe	0.756	0.846	0.08	0.14	2.688	0.010
Cho	mild	severe	1.783	1.550	0.21	0.31	2.995	0.005
Cr	mild	moderate	6.743	5.891	0.88	1.05	2.891	0.008
Cr	mild	severe	6.743	5.133	0.88	1.04	5.312	0.001
Cr	moderate	severe	5.891	5.133	1.05	1.04	2.872	0.016
NAA	mild	moderate	7.547	6.635	0.96	1.05	2.654	0.011
NAA	mild	severe	7.547	5.996	0.96	1.57	4.110	0.001

AC absolute concentration, SD standard deviation.

**Table 5:** The results of the paired-samples *t*-test comparing disease severity groups in the anterior cingulate cortex in the right hemisphere (Location 4).

AC (mM/kg)/Ratio	Groups		Mean		SD		*t*	*p*
Cho/Cr	mild	severe	1.152	1.286	0.11	0.13	3.540	0.001
Cho/NAA	mild	severe	0.760	0.839	0.09	0.16	2.126	0.040
Cho	mild	severe	1.829	1.560	0.30	0.33	2.726	0.009
Cr	mild	moderate	6.910	5.833	1.01	1.10	3.424	0.001
Cr	mild	severe	6.910	5.274	1.01	0.96	5.453	0.018
Cr	moderate	severe	5.833	5.274	1.10	0.96	2.135	0.037
NAA	mild	moderate	7.700	6.716	1.27	1.25	2.663	0.010
NAA	mild	severe	7.700	6.099	1.27	1.61	3.487	0.001
NAA	moderate	severe	6.716	6.099	1.25	1.61	1.725	0.090

AC absolute concentration, SD standard deviation.

**Table 6:** The results of the paired-samples *t*-test comparing disease severity groups in the deep frontal white matter in the left hemisphere (Location 5).

AC (mM/kg)/Ratio	Groups		Mean		SD		*t*	*p*
Cho/Cr	mild	severe	1.272	1.381	0.14	0.19	2.076	0.044
Cho/NAA	mild	severe	0.534	0.599	0.08	0.09	2.423	0.020
Cho	mild	severe	1.982	1.754	0.31	0.31	2.404	0.021
Cr	mild	severe	6.028	4.894	1.17	0.81	3.862	0.009
Cr	moderate	severe	5.482	4.894	1.02	0.81	2.496	0.015
NAA	mild	moderate	12.075	10.631	1.55	1.80	2.843	0.006
NAA	mild	severe	12.075	9.671	1.55	2.01	4.010	0.007

AC absolute concentration, SD standard deviation.

**Table 7:** The results of the paired-samples *t*-test comparing disease severity groups in the deep frontal white matter in the right hemisphere (Location 6).

AC (mM/kg)/Ratio	Groups		Mean		SD		*t*	*p*
Cho/NAA	mild	severe	0.523	0.575	0.07	0.07	2.328	0.025
Cho	mid	severe	2.082	1.773	0.35	0.29	3.216	0.002
Cho	moderate	severe	1.979	1.773	0.34	0.29	2.569	0.013
Cr	mild	moderate	6.398	5.822	0.88	0.97	2.078	0.043
Cr	mild	severe	6.398	5.133	0.88	0.66	5.472	0.019
Cr	moderate	severe	5.822	5.133	0.97	0.66	3.221	0.022
NAA	mild	moderate	12.848	11.631	1.21	1.79	2.910	0.006
NAA	mild	severe	12.848	10.054	1.21	1.71	5.896	0.002
NAA	moderate	severe	11.631	10.054	1.79	1.71	3.568	0.001

AC absolute concentration, SD standard deviation.

**Table 8:** The results of the paired-samples *t*-test comparing disease severity groups in the posterior cingulate cortex in the left hemisphere (Location 7).

AC (mM/kg)/Ratio	Groups		Mean		SD		*t*	*p*
Cho/Cr	moderate	severe	0.981	1.050	0.15	0.12	2.032	0.046
Cho/NAA	mild	severe	0.487	0.586	0.07	0.10	4.028	0.009
Cho/NAA	moderate	severe	0.528	0.586	0.08	0.10	2.621	0.011
Cr	mild	severe	7.199	6.141	1.47	1.10	2.764	0.028
Cr	moderate	severe	7.054	6.141	1.40	1.10	2.838	0.016
NAA	mild	severe	11.706	9.303	1.52	2.06	4.161	0.001
NAA	moderate	severe	10.895	9.303	2.07	2.06	3.053	0.007

AC absolute concentration, SD standard deviation.

**Table 9:** The results of the paired-samples *t*-test comparing disease severity groups in the posterior cingulate cortex in the right hemisphere (Location 8).

AC (mM/kg)/Ratio	Groups		Mean		SD		*t*	*p*
Cho/NAA	mild	severe	0.487	0.571	0.07	0.11	3.077	0.004
Cr	mild	moderate	7.248	6.600	0.79	1.05	2.253	0.029
Cr	mild	severe	7.248	6.017	0.79	1.00	4.300	0.001
Cr	moderate	severe	6.600	6.017	1.05	1.00	2.246	0.028
NAA	mild	severe	11.411	8.920	1.50	1.70	4.970	0.001
NAA	moderate	severe	10.454	8.920	2.38	1.70	2.878	0.005

AC absolute concentration, SD standard deviation.

**Table 10:** Characteristics of disease severity groups for each of the eight examined brain locations. Three absolute concentrations or ratios that contribute most significantly to the differences among severity groups according to the results of discriminant analysis are presented for each location. Additionally, the results of the Student’s *t*-test are included, with asterisks denoting statistically significant differences. The label *^2^ next to the highest values indicates a significant elevation compared to the other two groups (mid-level and lower), while *^1^ indicates significance solely compared to the group with lower values. If *^1^ is alongside the group with mid-level values, it denotes a significant elevation compared to the group with lower values.

Location	Metabolite Concentrations and Ratios	Groups Categorized According to Disease Severity			Contribution %
		Mild	Moderate	Severe	
**1**	Cr	6.469 *^2^	5.827 *^1^	5.233	50.333
	NAA	9.535 *^2^	8.902 *^1^	8.291	29.333
	Cho/NAA	0.666	0.701	0.739 *^1^	8.000
**2**	Cr	6.517 *^2^	5.973 *^1^	5.321	37.652
	NAA	10.075 *^2^	9.384 *^1^	8.502	28.340
	Cho/Cr	1.181	1.284 *^1^	1.390 *^2^	17.814
**3**	Cho	1.783 *^1^	1.651	1.550	42.105
	Cho/NAA	0.756	0.801	0.846 *^1^	24.342
	Cr	6.743 *^2^	5.891 *^1^	5.133	11.842
**4**	Cho/NAA	0.760	0.784	0.839 *^1^	38.767
	Cr	6.910 *^2^	5.833 *^1^	5.274	26.872
	NAA	7.700 *^2^	6.716 *^1^	6.099	14.978
**5**	Cho/NAA	0.534	0.571	0.599 *^1^	22.131
	NAA	12.075 *^2^	10.631 *^1^	9.671	20.765
	Cr	6.028 *^2^	5.482 *^1^	4.894	18.852
**6**	Cr	6.398 *^2^	5.822 *^1^	5.133	36.842
	NAA	12.848 *^2^	11.631 *^1^	10.054	22.601
	Cho	2.082 *^1^	1.979 *^1^	1.773	18.576
**7**	Cr	7.199 *^2^	7.054 *^1^	6.141	42.246
	Cho	2.033	2.051	1.982	26.738
	NAA	11.706 *^1^	10.895 *^1^	9.303	14.973
**8**	Cr	7.248 *^2^	6.600 *^1^	6.017	35.821
	Cho	2.069	2.055	1.887	32.537
	NAA	11.411 *^1^	10.454 *^1^	8.920	21.493

**Table 11:** Characteristics of disease severity groups across eight examined brain locations for the absolute concentration of Cr. The label ‘*^2^’ next to the highest values marks a significant elevation, as determined by the *t*-test, compared to the other two groups (mid-level and lower). Meanwhile, ‘*^1^’ denotes a significant increase only when compared to the group with the lower values. When ‘*^1^’ is assigned to mid-level values, it specifically indicates a significant increase relative to the lower group, according to the *t*-test results.

Location	Mild	Moderate	Severe	Contribution %
2	6.517 *^2^	5.973 *^1^	5.321	30.618
8	7.248 *^2^	6.600 *^1^	6.017	26.697
7	7.199 *^1^	7.054 *^1^	6.141	16.440
1	6.469 *^2^	5.827 *^1^	5.233	9.955
6	6.398 *^2^	5.822 *^1^	5.133	9.050
4	6.910 *^2^	5.833 *^1^	5.274	5.732
3	6.743 *^2^	5.891 *^1^	5.133	1.207
5	6.028 *^1^	5.482 *^1^	4.894	0.302
Homogeneity	15/17	23/36	20/28	
%	88.24	63.89	71.43	

**Table 12:** Characteristics of disease severity groups across eight examined brain locations for the absolute concentration of NAA. *^2^ Next to the highest values marks a significant elevation, as determined by the *t*-test, compared to the other two groups. *^1^ Denotes a significant increase only when compared to the group with the lower values.

Location	Mild	Moderate	Severe	Contribution %
6	12.848 *^2^	11.631 *^1^	10.054	50.368
5	12.075 *^2^	10.631 *^1^	9.671	16.544
2	10.075 *^2^	9.384 *^1^	8.502	13.971
1	9.535 *^2^	8.902 *^1^	8.291	5.882
4	7.700 *^2^	6.716 *^1^	6.099	5.515
8	11.411 *^1^	10.454 *^1^	8.920	4.779
3	7.547 *^2^	6.635	5.996	2.941
7	11.706 *^1^	10.895 *^1^	9.303	0.000
Homogeneity	13/17	19/36	20/28	
%	76.47	52.78	71.43	

## Data Availability

The human data supporting the findings of this study are not openly available due to privacy and sensitivity concerns. They are available from the corresponding author upon request.
